# The Research of Proteomics in Neurodegenerative Diseases Based on Bibliometric Analysis

**DOI:** 10.1002/brb3.70964

**Published:** 2025-11-26

**Authors:** Xiaoqiong An, Jun He, Wenfeng Yu, Zhenkui Ren

**Affiliations:** ^1^ Department of Laboratory Medicine The Second People's Hospital of Guizhou Province Guiyang China; ^2^ Guizhou Provincial Center For Clinical Laboratory Guiyang China; ^3^ Key Laboratory of Molecular Biology Guizhou Medical University Guiyang Guizhou China; ^4^ Key Laboratory of Human Brain Bank For Functions and Diseases of Department of Education of Guizhou Province Guizhou Medical University Guiyang Guizhou China; ^5^ Laboratory Department of People's Hospital of Southwest Guizhou Autonomous Prefecture Xingyi Guizhou China

**Keywords:** bibliometrix, CiteSpace, neurodegenerative disease, proteomics, VOSviewer

## Abstract

**Purpose:**

This study aims to provide a comprehensive bibliometric analysis of the literature on proteomics in neurodegenerative diseases in order to identify research trends, key contributors, and collaborative networks and to assess the field's development and potential future directions.

**Method:**

A bibliometric analysis was conducted using data from 3461 publications (articles and reviews) published between 2000 and 2024, retrieved from the Web of Science Core Collection (WoSCC) database. The data were analyzed using CiteSpace, VOSviewer, and the R package “bibliometrix” to analyze and visualize publication trends, international and institutional collaborations, influential authors, and research focus areas.

**Finding:**

This study uses bibliometric tools to visualize literature trends and collaborations. Analyzing 3461 publications from 2000 to 2024 shows a rise in publications, especially in the last 5 years, highlighting proteomics' importance in understanding diseases and finding therapies. The United States leads with 40% of publications, followed by China and Germany, with key institutions and authors identified.

**Conclusion:**

Despite bibliometric analysis limitations, our findings emphasize proteomics' role in neurodegenerative research, suggesting future studies should use diverse data sources for a comprehensive understanding.

## Graphical abstract

This bibliometric analysis of 3461 publications maps the evolving landscape of proteomics research in neurodegenerative diseases, highlighting key trends, collaborative networks, and emerging hotspots from 2000 to 2024.

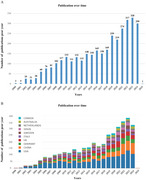

## 1 Introduction

Neurodegenerative disorders, including Alzheimer's and Parkinson's diseases, represent a substantial public health challenge due to their profound impact on individuals and society at large (Dawson et al. 2018). Characterized by the progressive degeneration of neurons, these conditions lead to cognitive deterioration, and reduced quality of life and impose significant economic burdens on healthcare systems and families (De Marchi et al. 2024; Luh and Bertolotti 2020). Despite advancements in pharmacological and non‐pharmacological therapies, current interventions have demonstrated limited efficacy in altering the progression of neurodegenerative diseases and are frequently associated with adverse side effects (Memon et al. 2020; Qin et al. 2022). Consequently, there is an imperative need for novel strategies to enhance early diagnostic capabilities and to develop more effective therapeutic interventions for these conditions.

Recent studies have underscored the potential of proteomics as a pivotal approach for investigating neurodegenerative diseases (Adam et al. 2024). Proteomics encompasses the comprehensive analysis of proteins and their functions, yielding critical insights into the molecular mechanisms underlying these conditions. Although proteomic methodologies have been successfully applied in other domains, such as cancer research (Cui et al. 2022), their application in the study of neurodegenerative diseases remains comparatively underexplored.

This study employs bibliometric analysis to evaluate the research landscape related to proteomics in neurodegenerative diseases. Utilizing tools such as VOSviewer, CiteSpace, and Bibliometrix, the study offers a comprehensive overview of the extant literature, elucidating trends, collaboration networks, and principal research hotspots. These quantitative methodologies are of growing importance as they facilitate the visualization and understanding of large data sets, thereby guiding researchers towards emerging areas of interest and potential opportunities for collaboration.

The primary objective of this research is to elucidate the application of proteomics in the investigation of neurodegenerative diseases, with a particular focus on identifying the leading contributors, institutions, and countries at the forefront of this domain. By examining the evolution of proteomic research pertaining to neurodegenerative disorders (Dugger and Dickson 2017), this study seeks to provide a comprehensive overview of the current state of the field and suggest potential directions for future research. By emphasizing the identification of critical biomarkers and therapeutic targets, this work aims to contribute to the development of more effective diagnostic and treatment strategies.

## 2 Materials and Methods

### 2.1 Data Retrieval and Collection

The Web of Science Core Collection (WoSCC) is a premier database for bibliometric analyses, renowned for its stringent selection criteria and comprehensive coverage across various academic disciplines. It serves as a cornerstone for global academic information retrieval, making it an ideal starting point for exploring the landscape of scholarly communication and impact. Thus, we are using this website as our search source.

**FIGURE 1:**
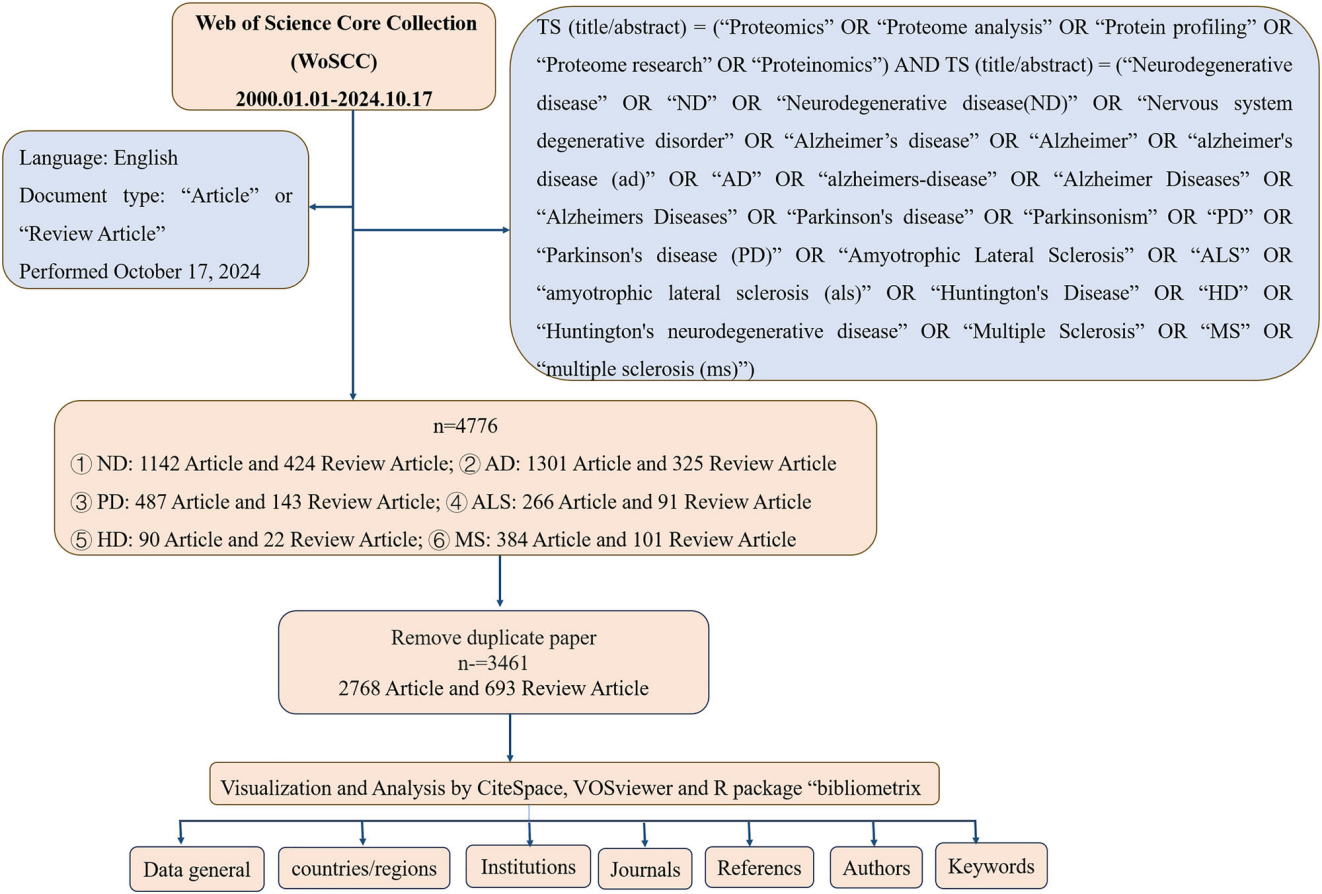
Flowchart of screening process.

Arches were conducted on October 17, 2024. According to the subject term “advanced search” method, the search terms are TS (title/abstract) = (“Proteomics” OR “Proteome analysis” OR “Protein profiling” OR “Proteome research” OR “Proteinomics”) AND TS (title/abstract) = (“Neurodegenerative disease” OR “ND” OR “Neurodegenerative disease (ND)” OR “Nervous system degenerative disorder” OR “Alzheimer's disease” OR “Alzheimer” OR “Alzheimer's disease (ad)” OR “AD” OR “Alzheimer's‐disease” OR “Alzheimer Diseases” OR “Alzheimer's Diseases” OR “Parkinson's disease” OR “Parkinsonism” OR “PD” OR “Parkinson's disease (PD)” OR “Amyotrophic Lateral Sclerosis” OR “ALS” OR “amyotrophic lateral sclerosis (als)” OR “Huntington's Disease” OR “HD” OR “Huntington's neurodegenerative disease” OR “Multiple Sclerosis” OR “MS” OR “multiple sclerosis (ms)”) AND Publication Date = (January 1, 2000 to October 17, 2024). Only articles and reviews written in English were selected, excluding meeting abstracts, editorial materials, proceedings papers, and other types of documents. We exported the search results in plain text file format and collected the full record and cited references of the documents, including authors, countries, institutions, references, keywords, journals, and other information. As shown in Figure 1, a detailed description of the screening process is provided.

### 2.2 Bibliometric and Visualized Analysis

The bibliometric analysis and visualization of publications retrieved from the WoSCC were conducted using VOSviewer (version 1.6.20), CiteSpace (version 6.3.R1), the Bibliometrix package, and an online platform for literature metrology analysis (https://bibliometric.com/) to perform bibliometric analyses (Jiang et al. 2023; Pei et al. 2022).

VOSviewer is a bibliometric analysis and the visualization of scientific knowledge software that aids researchers in analyzing and visualizing academic literature, knowledge networks, collaborative ties, and research trends (Arruda et al. 2022; van Eck and Waltman 2010). Its core functionalities encompass bibliometric analysis, co‐authorship network analysis, keyword co‐occurrence analysis, visualization, and thematic evolution analysis (Jiang et al. 2023; L. Zhang, Zheng, et al. 2024). Adjusting VOSviewer's parameters involves configuring various aspects such as network map visualization settings, label configurations, line settings, and color schemes (Wan et al. 2022). In this study, we used VOSviewer to conduct a visual analysis of the collaborative networks between countries, institutions, journals, and authors, as well as the co‐citation of keyword clusters.

CiteSpace, developed by Chen (2004), is a software tool capable of visually analyzing vast amounts of literature on topics, keywords, author affiliations, co‐authorship networks, journals, publication times, and citation counts. It supports data import from various databases such as Web of Science, Scopus, PubMed, and CNKI (China National Knowledge Infrastructure), and assists researchers in quickly understanding the development trajectory of a field, identifying research frontiers, and recognizing trends and developments (Yan et al. 2023). Through steps like setting up projects, importing data, selecting time ranges and node types, adjusting link strengths and selection criteria, and configuring network parameters, CiteSpace enables users to visualize and analyze literature data, identify research hotspots and trends, and export analysis results (Gao et al. 2022). In our study, we use CiteSpace to conduct a dual‐map analysis of journals, detect institutions, references, and keywords with the strongest citation bursts, generate clustering maps to visualize references and keywords, and create timeline visualizations to track the evolution of keywords over time.

Bibliometrix is an R tool for comprehensive scientific mapping and analysis, offering a full suite of tools from data collection to data visualization (Wan et al. 2022; Aria et al. 2021). It allows users to download data from various literature databases, such as Scopus and Web of Science, and perform analyses to generate dynamic charts (Y. Zhang, Jia, et al. 2024). We used Biblioshiny, a user‐friendly web interface for the R‐bibliometrix package, to visually display data analysis and social network graphs. Additionally, we leveraged R‐bibliometrix to perform frequency statistics on authors, journals, institutions, and countries, encompassing publication counts and citation numbers.

Through the collaborative use of these tools, we were able to delve into and display the academic landscape of proteomics research in the field of NDs from multiple angles, including research hotspots, collaboration networks, and knowledge evolution paths.

## 3 Results

### 3.1 Analysis of the Trend in Annual Publications

**FIGURE 2:**
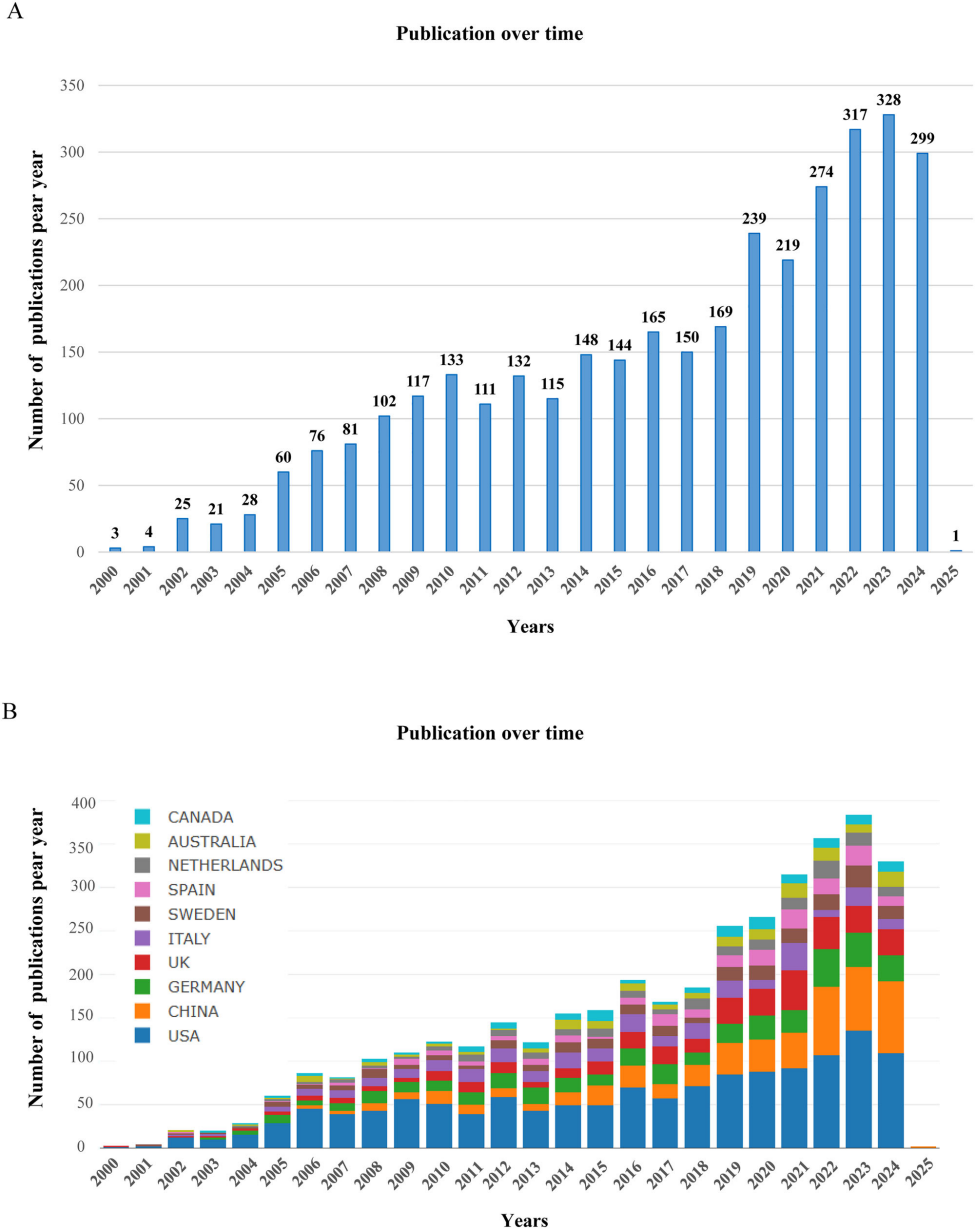
The number of publications published worldwide each year. (A) The publication volume and growth trends for proteomics research on neurodegenerative disease from 2000 to 2024. (B) Analysis of publication volume and growth trends in proteomics research on Neurodegenerative disease from 2000 to 2024 for the top 10 countries/regions, represented in a bar chart indicating annual publication counts.

A total of 3461 articles were included for further analysis based on publication time, type, language, and after removing duplicate documents. Figure 2A illustrates the temporal distribution of publications on proteomics research for ND. According to Figure 2A, the history of research can be divided into three phases: (i) 2010–2010: in the early stages, during the decade between 2000 and 2010, the number of publications increased greatly, climbing from just 3 to 133. (ii) 2011–2019: in the medium stage of development, there have been 111 publications in 2011 and 239 in 2019, and publication numbers are increasing slowly, but not steadily. (iii) 2020–2024: In the recent stage of development, the number of publications has increased from 219 in 2020 to 299 in 2024, consisting of 1437 articles published in the last 5 years, constituting 41% of all publications. Indicating the increasing application of proteomics in ND research highlights a powerful tool for early diagnosis, exploration of disease mechanisms, and the discovery of new therapeutic targets.

### 3.2 Analyses of Countries and Regions

**FIGURE 3:**
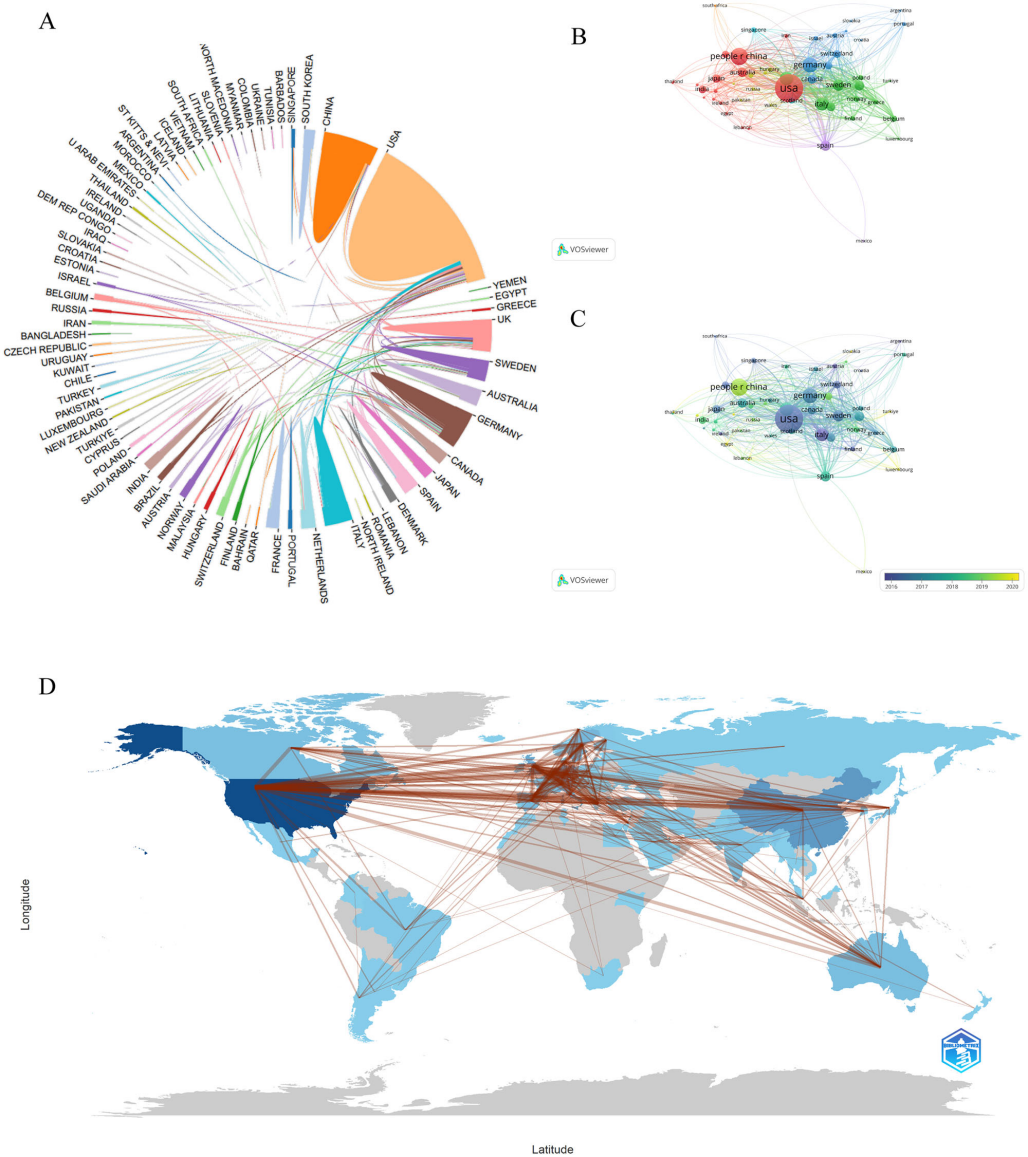
Country/regional contributions. (A) Bibliometrics' online analysis platform (http://bibliometric.com/) provides a collaborative network of countries and regions. Each area represents a country or region, and the larger the area, the more articles it has published. (B) Collaboration network analysis of the countries/regions. (C) Overlay visualization of co‐authorship countries or regions over time (years). Each country/region's name is sized to correspond with the number of articles in that country/region. The thickness of the curved connecting line represents the level of collaboration between countries/regions. Time intervals are represented by different colors inside the circle. (D) The geographical distribution of collaborations among countries and regions.

All proteomics‐related ND research publications were contributed by 79 countries/regions from 2000 to 2024. The past 20 years have seen many countries participating in research. As shown in Table 1, the top 10 countries/regions in terms of the number of publications are listed. The United States took the lead with a total of 1386 publications, accounting for 40% of the total, well ahead of other prominent nations like China, which had 534 articles (14%), and Germany, with 237 articles representing 11.6%. The United States leads with the highest number of citations, reaching 63,623, which signifies its dominance in the field. Although China ranks second in terms of publication volume, its citations per publication are comparatively low, only 17.24%, indicating a potential disparity between the quantity and impact of the research output. Notably, despite Sweden having a relatively small number of published articles, the average citations per article ranked second among the top 10 countries, suggesting that Swedish publications were of high quality. Figure 2B illustrates the annual publication trends among the top 10 countries, clearly showing that the United States has consistently held a prominent leadership position in the field of proteomics research concerning ND. Subsequently, we utilized an online bibliometric analysis platform (http://bibliometric.com/) to assess the significance of nations within cooperative networks. Figure 3A depicts the collaboration dynamics among various countries, with the United States exhibiting the most extensive cooperative efforts, particularly with Italy. Utilizing VOSviewer, we performed a visual analysis depicted in Figure 3B, where the size of the circles corresponds to the research contribution of each country in the field of proteomics and NDs. The lines connecting the circles signify collaborations between nations. Notably, the United States stands out for its substantial contributions. Figure 3C illustrates the average time it takes for researchers in different countries to initiate research in this field. It indicates that countries such as the United States, Italy, Iceland, and South Korea initiated their research before 2016. Other nations, including England and Spain, started their work around the mid‐2018 timeframe. In contrast, research in China, Denmark, Egypt, and several other countries commenced around 2020. Figure 3D provides a geographical visualization of countries' proteomics related to NDs, with red lines indicating collaboration between them.

**TABLE 1:** The top 10 countries/regions that publish research on proteomics in neurodegenerative disease.

Rank	Country/region	Publications	Citations	citations per publication	Total link strength	Links
1	USA	1386	63623	45.90	1023	49
2	People's Republic of China	534	9210	17.24	256	34
3	Germany	401	14208	35.43	659	43
4	England	326	10969	33.65	733	46
5	Italy	290	10967	37.82	375	37
6	Sweden	229	8671	37.86	539	39
7	Spain	185	4656	25.17	313	40
8	Netherlands	166	4962	29.89	367	35
9	Australia	144	5411	37.58	160	35
10	Canada	147	4542	30.90	144	35

### 3.3 An Analysis of the Major Institutions

**FIGURE 4:**
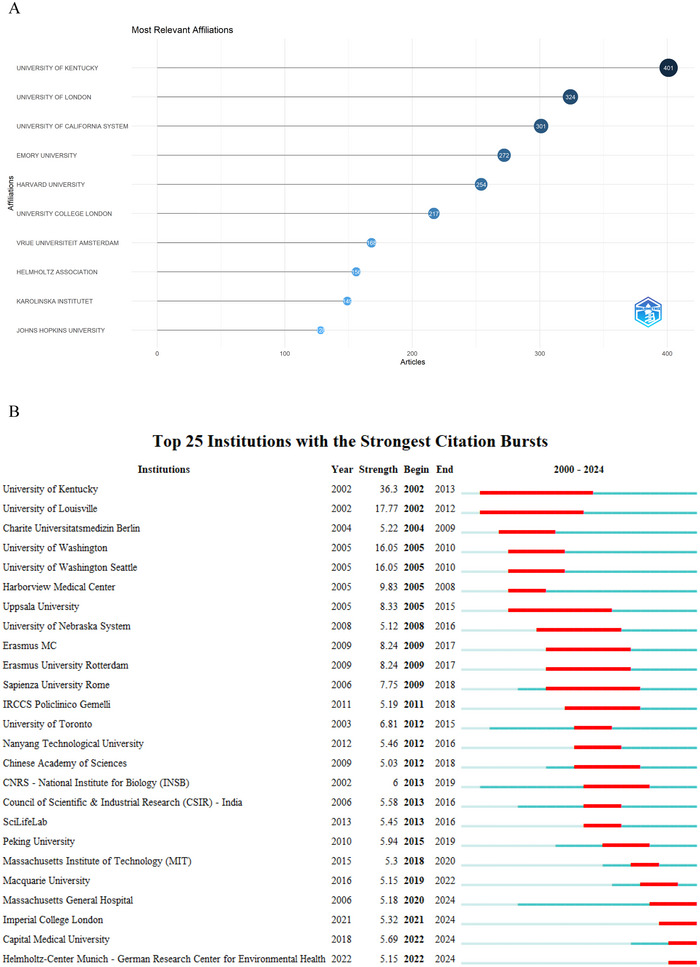
An analysis of institutions. (A) The top 10 most productive institutions in proteomics research related to neurodegenerative diseases. (B) Top 25 Institutions with the Strongest Citation Bursts.

**FIGURE 5:**
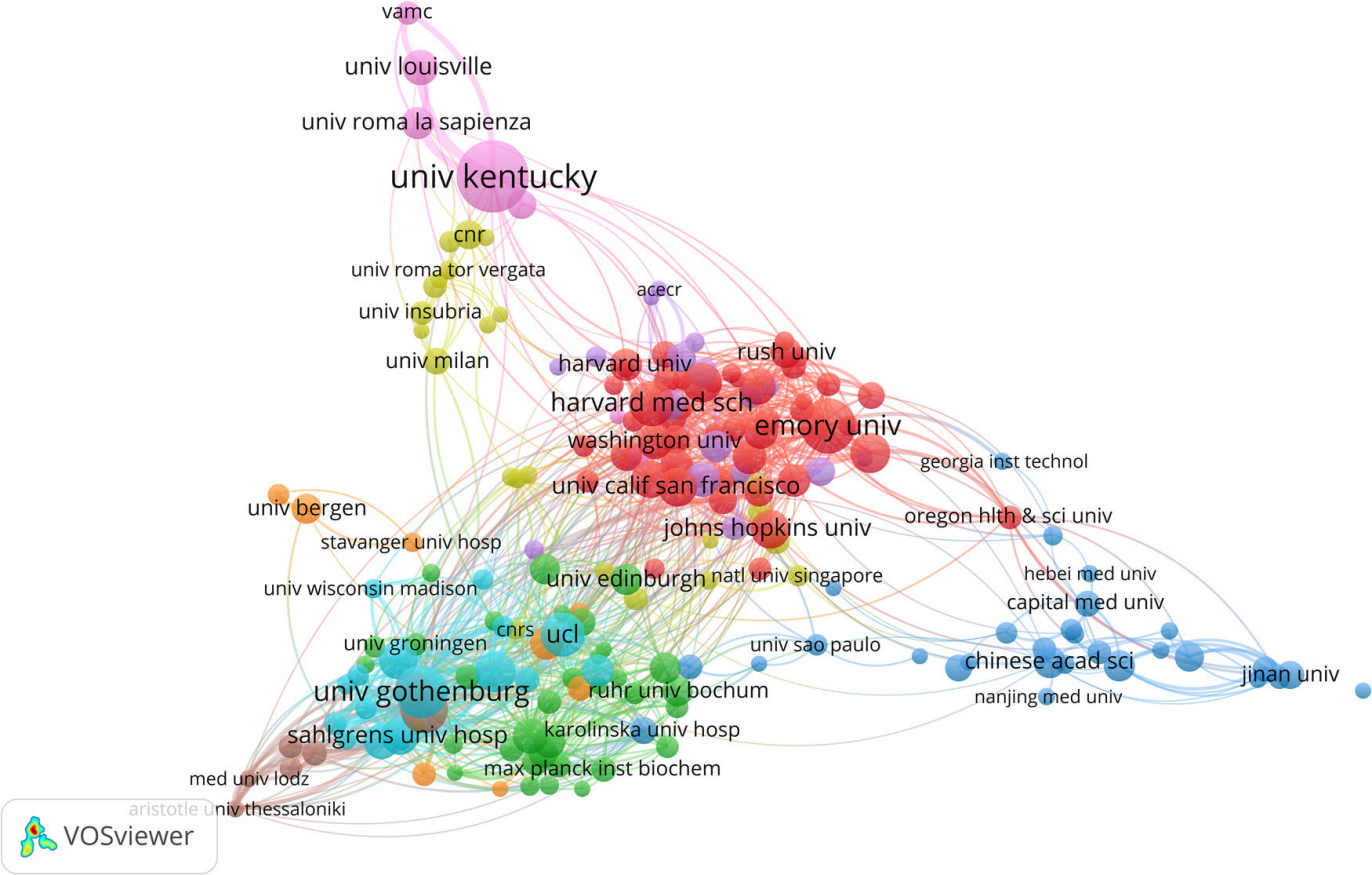
A visualization graph of collaborations between institutes in proteomics research related to neurodegenerative diseases, based on a minimum of 10 documents. Each node represents an institute, and the size of the node indicates the number of publications they produce, and the lines represent collaborations between institutions.

From 2000 to 2024, a cumulative total of 3670 institutions have been engaged in proteomics research concerning NDs. Based on a visualization analysis of the top 10 research institutions in Figure 4A, the University of Kentucky is the leading institution, with 401 publications, followed by the University of London with 324 articles and the University of California System with 301 articles. Notably, five of the top 10 institutions with the highest publication output in this field are based in the United States, underscoring the country's dominant role in ND proteomics research. Figure 4B, which uses red bars to denote citation bursts, shows that the University of Kentucky, the University of Louisville, the University of Washington, and the University of Washington Seattle were key contributors in the early years of this field, while Peking University, the Massachusetts Institute of Technology (MIT), and Macquarie University became active after 2015. Since 2020, Massachusetts General Hospital, Imperial College London, and Capital Medical University have also become increasingly engaged in this area of research. The VOSviewer's visualization analysis, as shown in Figure 5, reveals the intricate web of institutional cooperation in ND proteomics research. This intercontinental collaboration underscores the global nature of the field, with US and European institutions taking on pivotal roles in nurturing these collaborative efforts.

### 3.4 An Analysis of the Authors and Co‐Cited Authors

**FIGURE 6:**
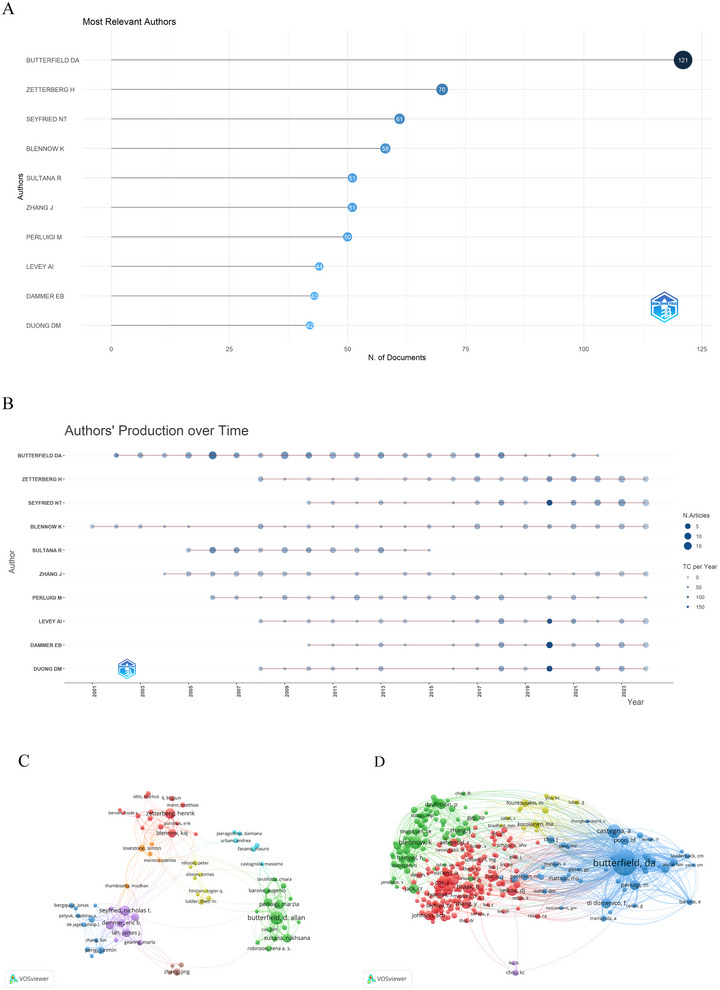
The visualization network map of co‐authorship and co‐citation of authors. (A) Top 10 most relevant authors in proteomics research related to neurodegenerative diseases. (B) Top 10 authors’ production over time. (C) Co‐authorship network visualization map of authors. The node size represents the number of publications by the author, similar colors represent connections within clusters, and the thickness of the line between the authors indicates the level of cooperation. (D) Co‐citation network visualization map of authors. Node sizes represent co‐citation association intensity.

The author's co‐occurrence analysis has pinpointed the key contributors in the field of ND proteomics research, as well as the extent of their collaborative ties. A total of 18,516 authors and 99,036 co‐cited authors were found in this analysis. Table 2 and Figure 6A display the top 10 prolific authors in the field of proteomics research for NDs. Among them, Butterfield Da, associated with the University of Kentucky, stands out as the preeminent figure. He leads in terms of publication count (*n* = 121), total citations (13,488 citations), and H‐index (68), a testament to his exceptional research productivity, the excellence of his scholarly contributions, and his extensive collaborative efforts with other researchers. Followed by Zetterberg H with 70 publications and Seyfried Nt with 61 publications. Figure 6B presents the annual publication and citation metrics for the top 10 authors, tracking their scholarly contributions and influence across time.

**TABLE 2:** Top 10 authors with the most publications and top 10 authors with the most local citations.

Rank	Author	Count	h_index	Total citations	Co‐cited author	Total citations
1	Butterfield Da	121	68	13488	Butterfield Da	2588
2	Zetterberg H	70	28	2289	Klein Jb	1211
3	Seyfried Nt	61	27	2574	Pierce Wm	1193
4	Blennow K	58	28	2758	Sultana R	1103
5	Sultana R	51	37	5744	Markesbery Wr	960
6	Zhang J	51	27	2108	Perluigi M	712
7	Perluigi M	50	34	3647	Lah Jj	684
8	Levey Ai	44	28	2462	Castegna A	676
9	Dammer Eb	43	24	2269	Duong Dm	668
10	Duong Dm	42	27	2576	Zhang J	650

Co‐authorship networks are depicted in Figure 6C, with the unit of analysis being the author, and the threshold for constructing a network being set to a minimum of ten documents per author. To be part of this network, an author must have contributed at least 10 papers in the domain of ND proteomics, either as the primary or a co‐author. Among 18,516 authors, 100 qualified based on this threshold and are represented in the network with connections to other authors. In terms of authorship, the largest network connection included 71 entries distributed among eight clusters. The author with the most significant node in the network is Butterfield Da.

Co‐citation analysis reveals relationships between two authors or papers that are cited together by a third author or paper. As shown in Figure 6D, authors who have been co‐cited more than 50 times exhibit co‐citation relationships. It mainly shows five clusters, highlighting the authors with significant influence in the field of ND proteomics research. Table 2 highlights the top 10 most cited authors. Notably, 87 authors have had their articles cited over 100 times, signifying their research holds considerable prestige and influence in the field. Table 2 specifically shows that among the top 10 co‐citation authors, Butterfield Da tops the list with 2588 citations, followed by J.B. Klein with 1211 citations, and W.M. Pierce with 1193 citations.

### 3.5 An Analysis of the Journals and Co‐Cited Journals

**FIGURE 7:**
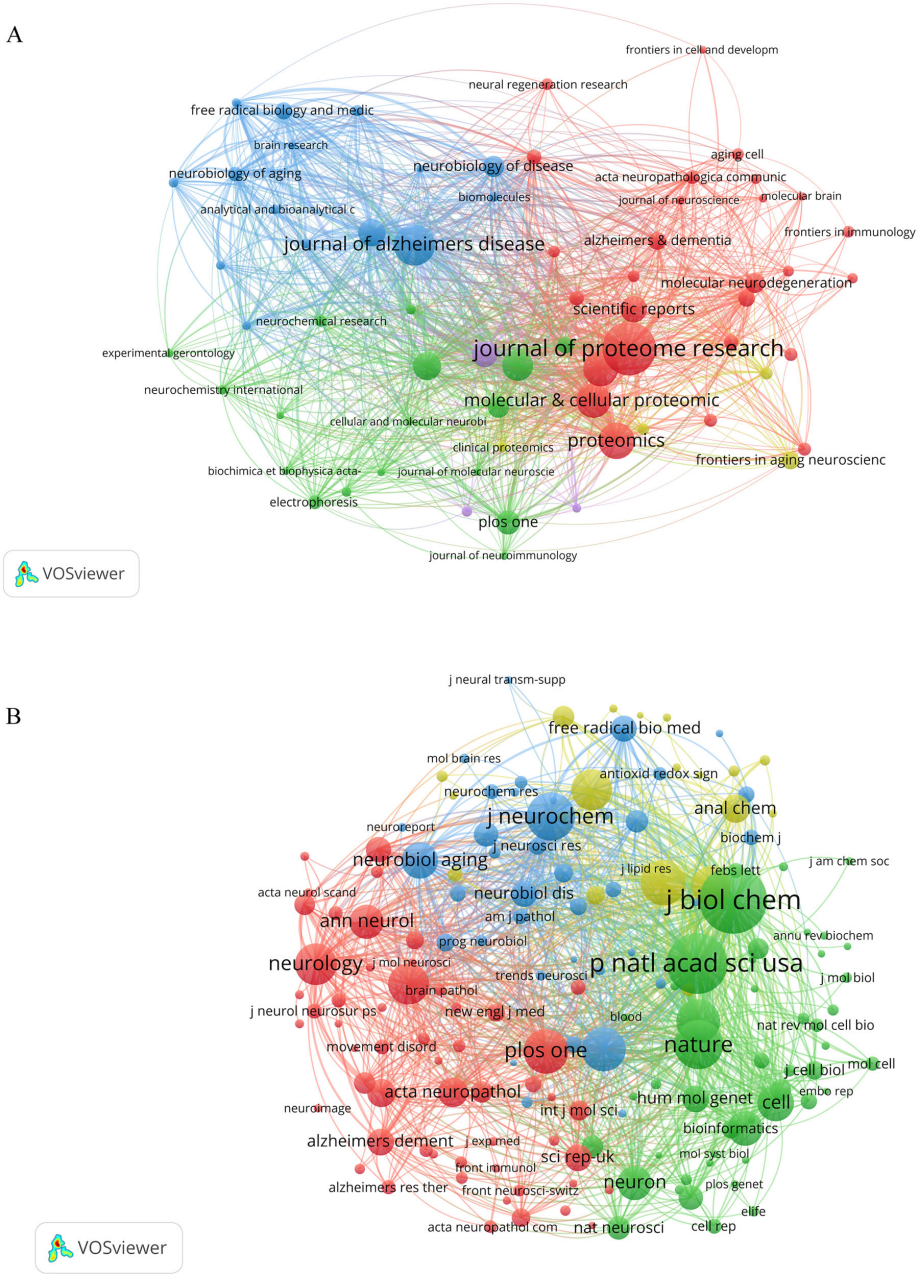
The network co‐occurrence diagram of journals and co‐cited journals. (A) A network visualization of the citation journal analysis was generated by VOSviewer. Node size shows co‐occurrence frequency, links reflect the co‐occurrence relationships between journals, and color indicates cluster class. (B) A network visualization of the co‐citation journal analysis was generated by VOSviewer. Each node represents a journal that is co‐cited, and each color represents a cluster. The links between nodes represent the journals that are co‐cited.

A total of 3461 articles related to ND proteomics research have been published across 831 distinct journals from 2000 to 2024. We used VOSviewer to visualize the co‐occurrence and co‐citation relationships within the journal landscape of proteomics research on NDs. Figure 7A displays the network of journals based on their co‐occurrence, while Figure 7B illustrates the connections between journals that frequently cite each other. From these analyses, we identified and ranked the top 10 journals and their co‐cited journals associated with proteomics research on NDs, as summarized in Table 3. Among them, the *Journal of Proteome Research* (IF = 3.8, Q1) is the leading journal, having published the most papers (149, 4.31%). It focuses on proteomics research, including the proteomic analysis of NDs, the identification of biomarkers, and the study of disease mechanisms. Followed by the *Journal of Alzheimer's Diseases* (103, 2.98%), *Proteomics* (87, 2.51%), and the *International Journal of Molecular Sciences* (81, 2.34%). The *Journal of Alzheimer's Disease* (IF = 3.4, Q2) is an international multidisciplinary journal dedicated to advancing understanding of the etiology, pathogenesis, epidemiology, genetics, behavior, treatment, and psychology of Alzheimer's Disease. *Proteomics* (IF = 3.4, Q2) mainly focuses on systems biology and proteomics research, including the field of NDs. It publishes studies on the proteomic analysis of these diseases and how proteomic techniques reveal their complex biology. The *International Journal of Molecular Sciences* (IF = 4.9, Q2) is a multidisciplinary journal of molecular sciences, including research on the molecular mechanisms of NDs. It publishes studies on molecular subtypes, RNA sequencing data, and the exploration of new mechanisms and targets for these diseases. These journals play a significant role in the field of ND research, covering a broad range of studies from basic science to clinical applications, including pathological mechanisms, biomarker discovery, treatment strategies, and molecular diagnostics. By publishing high‐quality original research papers and reviews, these journals provide valuable knowledge and insights into the field of ND research.

**TABLE 3:** The top 10 journals and the co‐cited journals that published documents on proteomics research related to neurodegenerative diseases.

Rank	Sources	Articles	Citations	Total link strength	IF (2023)	JCR (2023)	Co‐cited Journal	Co‐citations	Total link strength	IF(2023)	JCR (2023)
1	*Journal of Proteome Research*	149	5347	795	3.8	Q1	*Journal of Biological Chemistry*	7234	518923	4	Q2
2	*Journal of Alzheimer's Disease*	103	4410	822	3.4	Q2	*Proceedings of the National Academy of Sciences of the United States of America*	6294	449296	9.4	Q1
3	*Proteomics*	87	2681	413	3.4	Q2	*Nature*	4652	336800	50.5	Q1
4	*International Journal of Molecular Sciences*	81	943	310	4.9	Q2	*Journal of Neurochemistry*	4488	344507	4.2	Q2
5	*Molecular & Cellular Proteomics*	79	3473	379	6.1	Q1	*Journal of Proteome Research*	4093	271780	3.8	Q1
6	*Journal Of Proteomics*	69	2506	443	2.8	Q2	*Plos One*	3992	279762	2.9	Q2
7	*Expert Review of Proteomics*	61	1333	516	3.8	Q1	*Science*	3961	277085	44.8	Q1
8	*Journal of Neurochemistry*	58	3055	673	4.2	Q2	*The Journal of Neuroscience*	3892	292264	4.4	Q1
9	*Scientific Reports*	56	1381	190	3.8	Q1	*Neurology*	3662	265001	8.4	Q1
10	*Proteomics Clinical Applications*	51	1521	480	2.1	Q3	*Journal of Alzheimer's Disease*	3601	277085	3.4	Q2

**FIGURE 8:**
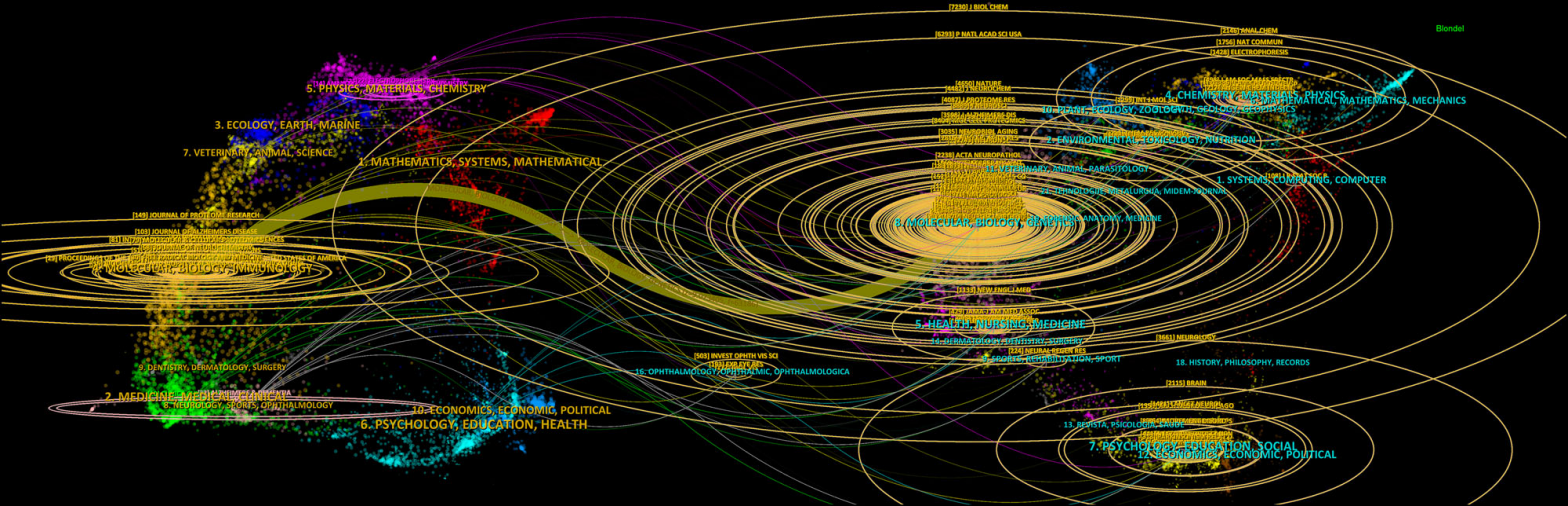
Dual‐map overlay of journals in the field of proteomics application in neurodegenerative diseases from 2000 to 2024. Citing journals are displayed on the left side of the chart, while cited journals are shown on the right. Citations are represented by colored paths.

The significance of a journal's impact is often gauged by its co‐citation frequency, indicating its influence within a specific research domain. The top 10 co‐cited journals ranked by number of citations are listed in Table 3. Among them, the *Journal of Biological Chemistry* (IF = 4.0, Q2) leads with the highest co‐citation (7234 citations), followed by the *National Academy of Sciences USA* (IF = 9.4, Q1) with 6294 citations, and *Nature* (IF = 50.4, Q1) with 4652 citations. Additionally, we employed CiteSpace to create a dual map overlay of journals, which visually represents the distribution of academic journals in the field (Guan et al. 2023). As illustrated in Figure 8, the left side of the graph depicts the citing journals, while the right side represents the cited journals, effectively highlighting the inter‐journal relationships within the academic landscape. The colored line represents the primary citation pathway, indicating that research on proteomics related to NDs, published in molecular biology and immunology journals (*z* = 9.073), is predominantly cited by publications in molecular biology and genetics journals.

### 3.6 An Analysis of Cited References and Co‐Cited References

**FIGURE 9:**
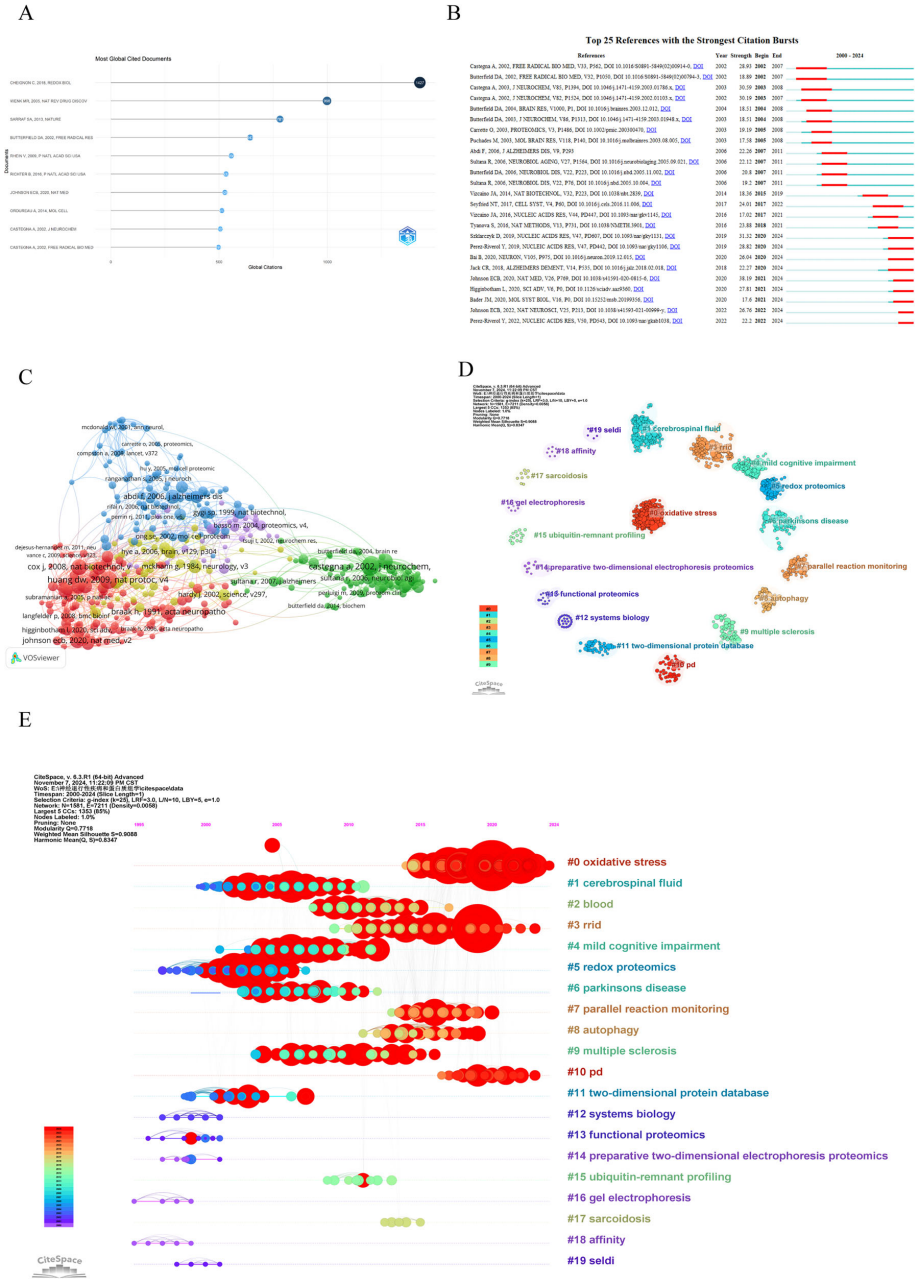
An analysis of cited references and co‐cited references. (A) Top 10 most globally cited documents in proteomics related to neurodegenerative disease research. (B) Top 25 references with the strongest citation bursts based on CiteSpace. Citation burst strength is a metric that quantifies the intensity of a burst during its duration. Citations with higher strengths indicate a greater impact or influence in the field over a relatively short period of time. (C) The network map shows the co‐citation analysis of references with more than 25 citations. (D) Cluster analysis of co‐cited literature network. Colors are used to differentiate between clusters, with similar‐colored nodes suggesting a higher degree of thematic or research area coherence. (E) Timeline visualization of co‐cited references cluster analysis. It depicts a timeline visualization of the cluster analysis results for co‐cited references spanning from January 1, 2000 to October 17, 2024.

By analyzing citations and co‐citations in the literature, we can gain a better understanding of the field's seminal research and chart a course for future research. Table 4 and Figure 9A present the top 10 most‐cited articles in the field of proteomics applications in NDs, with citation counts ranging from 497 to 1427. The article “Oxidative Stress and the Amyloid Beta Peptide in Alzheimer's Disease (Cheignon et al. 2018)” published in 2018 in “Redox Biology” leads with the highest number of citations (1427). It is followed by the article “The emerging field of lipidomics (Wenk 2005)” published in 2005 in “Nature Reviews Drug Discovery,” which has a citation count of 998, and “Landscape of the PARKIN‐dependent ubiquitylome in response to mitochondrial depolarization (Sarraf et al. 2013)” published in 2013 in “Nature,” which has a citation count of 781. The top 10 articles highlight the pivotal role of proteomics in uncovering the molecular mechanisms of NDs, discovering biomarkers, and developing new therapeutic strategies.

**TABLE 4:** The top 10 most‐cited documents globally.

Rank	Literature	Total citations	Author	Year	Journal	IF (2023)	JCR (2023)	DOI
1	Oxidative stress and the amyloid beta peptide in Alzheimer's disease	1427	C. Cheignon	2018	Redox Biology	10.1	Q1	10.1016/j.redox.2017.10.014
2	The emerging field of lipidomics	998	Markus R. Wenk	2005	Nature Reviews Drug Discovery	122.7	Q1	10.1038/nrd1776
3	Landscape of the PARKIN‐dependent ubiquitylome in response to mitochondrial depolarization	781	Shireen A. Sarraf	2013	Nature	50.5	Q1	10.1038/nature12043
4	Amyloid beta‐peptide (1‐42)‐induced oxidative stress and neurotoxicity: implications for neurodegeneration in Alzheimer's disease brain. A review	643	D Allan Butterfield	2002	Free Radical Research	2.6	Q2	10.1080/1071576021000049890
5	Amyloid‐beta and tau synergistically impair the oxidative phosphorylation system in triple transgenic Alzheimer's disease mice	556	Virginie Rhein	2009	Proceedings of the National Academy of Sciences of the United States of America	9.4	Q1	10.1073/pnas.0905529106
6	Phosphorylation of OPTN by TBK1 enhances its binding to Ub chains and promotes selective autophagy of damaged mitochondria	532	Benjamin Richter	2016	Proceedings of the National Academy of Sciences of the United States of America	9.4	Q1	10.1073/pnas.1523926113
7	Large‐scale proteomic analysis of Alzheimer's disease brain and cerebrospinal fluid reveals early changes in energy metabolism associated with microglia and astrocyte activation	526	Erik C. B. Johnson	2010	Nature Medicine	58.7	Q1	10.1038/s41591‐020‐0815‐6
8	Quantitative proteomics reveal a feedforward mechanism for mitochondrial PARKIN translocation and ubiquitin chain synthesis	513	Alban Ordureau	2014	Molecular Cell	14.5	Q1	10.1016/j.molcel.2014.09.007
9	Proteomic identification of oxidatively modified proteins in Alzheimer's disease brain. Part II: dihydropyrimidinase‐related protein 2, alpha‐enolase and heat shock cognate 71	505	Alessandra Castegna	2002	Journal of Neurochemistry	4.2	Q2	10.1046/j.1471‐4159.2002.01103.x
10	Proteomic identification of oxidatively modified proteins in Alzheimer's disease brain. Part I: creatine kinase BB, glutamine synthase, and ubiquitin carboxy‐terminal hydrolase L‐1	497	Alessandra Castegna	2002	Free Radical Biology and Medicine	7.1	Q1	10.1016/S0891‐5849(02)00914‐0

Citation emergence denotes a sharp rise in citation rates over a brief period, indicating temporal research trends and shifts in scholarly focus, and is utilized to assess the evolving dynamics and trajectories of research interests (Urlings et al. 2021). We utilized CiteSpace for a citation burst analysis on the clustered data, effectively mapping the temporal citation trends of key documents. Figure 9B shows the top 25 references with the strongest citation bursts, among which, the article titled “STRING v11: protein‐protein association networks with increased coverage, supporting functional discovery in genome‐wide experimental datasets (Szklarczyk et al. 2019),” published in 2019, ranked first (strength = 31.32). It emphasizes the role of the STRING database in the analysis of protein‐protein interaction networks, especially in the functional discovery of genome‐wide experimental datasets. “Proteomic identification of nitrated proteins in Alzheimer's disease brain (Castegna et al. 2003) ” and “Proteomic identification of oxidatively modified proteins in Alzheimer's disease brain. Part II: dihydropyrimidinase‐related protein 2, alpha‐enolase, and heat shock cognate 71 (Castegna, Aksenov, Thongboonkerd, et al. 2002)” by Alessandra Castegna, published in the Journal of Neurochemistry, also had a high burst, with strengths of 30.59 and 30.20, respectively. There are key clusters and citation bursts within the field of proteomics applications in NDs that can be seen in these figures, which provide insight into how topics have evolved over time.

In addition, co‐cited references were analyzed by VOSviewer to illustrate the most influential works. As shown in Figure 9C, we analyzed 394 references that were co‐cited in more than 25 citations from 160,262 references. It primarily displays five clusters, each represented by different colors for the various reference groups. The first cluster, colored red, comprises 191 references. The second, in green, consists of 119 references. The blue cluster contains 117 references, the yellow one includes 102 references, and the purple cluster includes 49 references. Table 5 presents the top 10 references that have received the highest number of citations. The article with the largest number of citations is “Proteomic identification of oxidatively modified proteins in Alzheimer's disease brain. Part II: dihydropyrimidinase‐related protein 2, alpha‐enolase, and heat shock cognate 71 (Castegna et al. 2002)” (156 citations), followed by “Systematic and integrative analysis of large gene lists using DAVID bioinformatics resources (D. W. Huang et al. 2009)” (154 citations). The third most co‐cited article is “Proteomic identification of oxidatively modified proteins in Alzheimer's disease brain. Part I: creatine kinase BB, glutamine synthase, and ubiquitin carboxy‐terminal hydrolase L‐1 (Castegna, Aksenov, Thongboonkerd, et al. 2002) ” (140 citations).

**TABLE 5:** The top 10 most co‐cited references.

Rank	Cited References	Co‐cited counts	Author	Year	Journal	IF (2023)	JCR (2023)	DOI
1	Proteomic identification of oxidatively modified proteins in Alzheimer's disease brain. Part II: dihydropyrimidinase‐related protein 2, alpha‐enolase and heat shock cognate 71	156	Alessandra Castegna	2002	Journal of Neurochemistry	4.2	Q2	10.1046/J.1471‐4159.2002.01103.X
2	Systematic and integrative analysis of large gene lists using DAVID bioinformatics resources	154	Da Wei Huang	2009	Nature Protocols	13.1	Q1	10.1038/NPROT.2008.211
3	Proteomic identification of oxidatively modified proteins in Alzheimer's disease brain. Part I: creatine kinase BB, glutamine synthase, and ubiquitin carboxy‐terminal hydrolase L‐1	140	Alessandra Castegna	2002	Free Radical Biology and Medicine	7.1	Q1	10.1016/S0891‐5849(02)00914‐0
4	MaxQuant enables high peptide identification rates, individualized p.p.b.‐range mass accuracies and proteome‐wide protein quantification	134	Jürgen Cox	2008	Nature Biotechnology	33.1	Q1	10.1038/NBT.1511
5	Neuropathological stageing of Alzheimer‐related changes	125	H. Braak	1991	Acta Neuropathologica	9.3	Q1	10.1007/BF00308809
6	Proteomic identification of nitrated proteins in Alzheimer's disease brain	122	Alessandra Castegna	2003	Journal of Neurochemistry	4.2	Q2	10.1046/J.1471‐4159.2003.01786.X
7	Large‐scale proteomic analysis of Alzheimer's disease brain and cerebrospinal fluid reveals early changes in energy metabolism associated with microglia and astrocyte activation	116	Erik C B Johnson	2020	Nature Medicine	58.7	Q1	10.1038/S41591‐020‐0815‐6
8	Detection of biomarkers with a multiplex quantitative proteomic platform in cerebrospinal fluid of patients with neurodegenerative disorders	115	Fadi Abdi	2006	Journal of Alzheimers Disease	3.4	Q2	10.3233/jad‐2006‐9309
9	The Perseus computational platform for comprehensive analysis of (prote)omics data	115	Stefka Tyanova	2016	Nature Methods	36.1	Q1	10.1038/NMETH.3901
10	The STRING database in 2023: protein‐protein association networks and functional enrichment analyses for any sequenced genome of interest	108	Damian Szklarczyk	2023	Nucleic Acids Research	16.6	Q1	10.1093/NAR/GKAC1000

When delving into the interconnections among academic literature, co‐citation analysis reveals a complex network composed of multiple clusters. In this network, each node symbolizes an independent reference, and the size of each node visually represents the number of citations it has received—the larger the node, the greater its influence in the academic community. As depicted in Figure 9D,E, references that are frequently co‐cited are organized into 20 major clusters, each distinguished by a unique color. In the timeline view, nodes positioned on the left side correspond to older references, while those on the right side are more recent. Nodes aligned horizontally belong to the same cluster, which is labeled with a number sign (#) on the right. This view highlights several emerging research hotspots, including “oxidative stress” (#0), “cerebrospinal fluid” (#1), “blood” (#2), “Rrid” (#3), “mild cognitive impairment” (#4), “redox proteomics” (#5), “Parkinsons disease” (#6), “parallel reaction monitoring” (#7), “autophagy” (#8), “multiple sclerosis” (#9), “pd” (#10), “two‐dimensional protein database” (#11), “systems biology” (#12), “functional proteomics” (#13), “preparative two‐dimensional electrophoresis proteomics” (#14), “ubiquitin‐remnant profiling” (#15), “gel electrophoresis” (#16), “sarcoidosis” (#17), “affinity” (#18), and “seldi” (#19). These topics, clearly identified by clusters of different colors in the timeline view, provide an important perspective for our in‐depth understanding of the application of proteomics in the research of NDs.

### 3.7 Analysis of Keyword Hotspots and Word Frequencies

**FIGURE 10:**
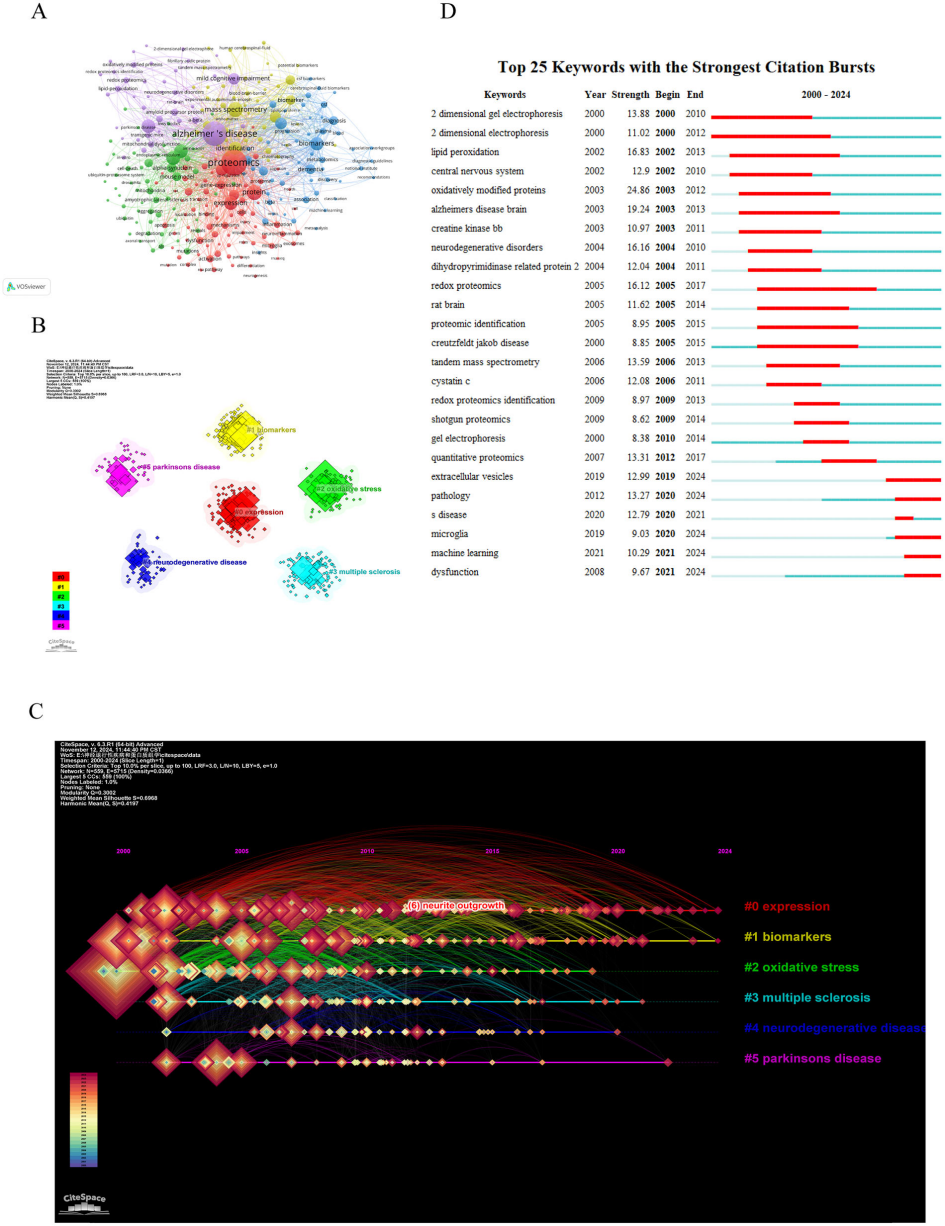
Keywords clustering and bursting. (A) Co‐occurrence network map of keywords with at least 20 occurrences. Different colors indicated diverse research clusters. (B) Cluster Analysis of keywords. (C) Timeline distribution of cluster analysis of the keyword. Colors showed the evolution of the keyword over time. (D)Top 25 keywords with the strongest citation bursts based on CiteSpace.

Keywords serve as a distillation of the core concepts within a research domain, and the analysis of their co‐occurrence provides valuable insights into the interconnectedness of key terms in the field of proteomics related to NDs. Through our analysis, we identified 12,936 relevant keywords at the nexus of proteomics and NDs. Table 6 presents the top 10 most frequently occurring keywords, along with their cumulative link strength, providing a quantitative view of the dominant themes and concepts in current research. Among the most frequent words were “proteomics” (1610 occurrences), followed by “Alzheimer's disease” (1422 occurrences) and “protein” (543 occurrences). Utilizing VOSviewer, we performed a co‐occurrence analysis that filtered out 277 keywords from the total, selecting only those that co‐occurred more than 20 times, ensuring our focus was on the most significant and frequently discussed topics. Figure 10A displays the keyword network, with the largest cluster being Cluster 1 (red), comprising 78 keywords such as “proteomics,” “protein,” and “expression.” Cluster 2 (green) contains 54 keywords, including terms like “Parkinson's disease,” “neurodegenerative diseases,” and “alpha‐synuclein.” Cluster 3 (blue) encompasses 53 keywords, primarily “biomarkers,” “tau,” and “cerebrospinal fluid.” Cluster 4 (yellow) consists of 52 keywords, with a focus on “mass spectrometry,” “identification,” and “multiple sclerosis.” Lastly, Cluster 5 (purple) comprises 40 keywords, highlighting “Alzheimer's disease,” “oxidative stress,” and “mild cognitive impairment.” Keywords like “proteomics,” “Alzheimer's disease,” “protein,” “mass spectrometry,” and “neurodegenerative diseases” emerged as the most frequently co‐occurring, indicating their centrality and strong linkages to other keywords in the field.

**TABLE 6:** The top 10 most frequently occurring keywords.

Rank	Keyword	Occurrences	Total link strength	Rank	Keyword	Occurrences	Total link strength
1	Proteomics	1610	1603	6	Expression	452	452
2	Alzheimer's disease	1422	1410	7	Parkinson's disease	446	446
3	Protein	543	540	8	Oxidative stress	433	433
4	Mass spectrometry	533	525	9	Biomarkers	431	427
5	Neurodegenerative diseases	466	462	10	Identification	397	395

We also utilized CiteSpace to visualize the co‐occurrence network of keywords, which revealed six distinct clusters within the literature, as depicted in Figure 10B,C. These clusters are centered around key themes: “expression”(cluster #0), “biomarkers”(cluster #1), “oxidative stress”(cluster #2), “multiple sclerosis” (cluster #3), “neurodegenerative diseases” (cluster #4) and “Parkinson's disease” (cluster #5). These results highlight the key research themes in the field, and the timeline graph shows that from 2000 to 2024, a multitude of researchers have conducted extensive and systematic studies on proteomics related to NDs, exploring the subject from diverse perspectives. Research hotspots are represented by larger thematic clusters, and the timeline illustrates the evolution of these hotspots over time. This visual analysis method helps us understand the co‐occurrence relationships between keywords, reveals current research hotspots, and tracks changes in research trends.

Burst keywords, which indicate a significant increase in the usage of specific terms within certain periods, provide insight into the dynamic research priorities and emerging trends in academia. Figure 10D highlights the top 25 keywords with the strongest citation bursts in the field of ND proteomics. Among them, “oxidatively modified proteins” exhibited the strongest burst (strength = 24.86) from 2003 to 2012, followed by “Alzheimer's disease brain” (strength = 19.24) from 2003 to 2013, “lipid peroxidation” (strength = 16.83) from 2002 to 2013, and “neurodegenerative disorders” (strength = 16.16) from 2004 to 2010. More recent bursts are observed for keywords such as “extracellular vesicles” (EV) (2019–2024), “pathology” (2020–2024), and “machine learning” (2021–2024), indicating the current and future directions of research interest.

## 4 Discussion

The analysis of publication trends in proteomics related to NDs shows a significant increase in scholarly output over the past two decades. From 2000 to 2010, the number of published articles rose dramatically from just three to 133. This upward trend continued, though at a slower rate, with publication numbers fluctuating between 111 and 239 from 2011 to 2019. The most notable growth occurred between 2020 and 2024, when the number of articles jumped from 219 in 2020 to an estimated 299 by 2024, resulting in a total of 1437 articles published in the last five years, which accounts for approximately 41% of all publications during this period. This surge highlights the increasing recognition of proteomics as an essential tool for unraveling the complex mechanisms behind NDs like Alzheimer's and PD, as well as for discovering potential biomarkers that can aid in early diagnosis and the development of targeted therapies (Raghunathan et al. 2022; Mayo et al. 2021; Karayel et al. 2022). Notably, recent advances in proteomic studies have increasingly focused on the role of key proteins such as tau in AD and alpha‐synuclein in PD, which are established as critical biomarkers and therapeutic targets (Raghunathan et al. 2022; Bai et al. 2021; Zhu et al. 2024). Furthermore, signaling pathways, including oxidative stress, autophagy, and the ubiquitin‐proteasome system, have emerged as central to understanding disease pathogenesis, with proteomics providing insights into their dysregulation and interactions (Perluigi et al. 2024; Zhou et al., 2024; Liu et al. 2025).

The geographical distribution of research reveals that the United States is the leading contributor in this field, with 1386 publications, accounting for 40% of the total output. Following the United States, China and Germany have made significant contributions as well, with 534 and 401 publications, respectively. This notable difference in publication volume may be indicative of varying levels of funding, research infrastructure, and collaborative networks among different countries. These trends underscore the critical role of international cooperation in advancing proteomic research (Kalita et al. 2018; Stoevesandt and Taussig, 2012; Okuda et al. 2017). Meanwhile, EVs have emerged as promising tools for early diagnosis due to their ability to cross the blood‐brain barrier and transport disease‐specific proteins (Ramos‐Zaldívar et al. 2022). Current research is increasingly centered on the identification of novel biomarkers derived from EVs, which possess distinctive disease‐specific proteomic signatures that offer innovative solutions for the early detection and dynamic monitoring of NDs (Vinaiphat and Sze 2022; Lee et al. 2024). Utilizing advanced proteomic technologies to examine the molecular profiles conveyed by EVs, researchers have identified several potential diagnostic markers, including Aβ, tau, ANXA5, VGF, and GPM6A (Q. Zhang et al. 2021; Muraoka et al. 2020). Recent evidence indicates that these proteins not only contribute significantly to the development of hallmark pathological structures in Alzheimer's disease, such as amyloid plaques and neurofibrillary tangles, but also play a crucial role in oxidative stress‐mediated neurodegenerative processes (Butterfield and Dalle‐Donne 2012; Butterfield et al. 2006; Butterfield and Boyd‐Kimball 2019; Sultana et al. 2009).

A closer look at the contributions of various institutions shows that American universities are crucial in advancing neurodegenerative proteomics research. For example, Kentucky University stands out with 401 published articles, while the University of London and the University of California system follow with 324 and 301 publications, respectively. This indicates that a limited number of institutions are making significant strides in fostering innovation and generating knowledge in this field. Additionally, the competitive environment among these research institutions can encourage collaborative projects and the sharing of knowledge, which are vital for addressing the complex challenges associated with NDs (Chou et al. 2020; Eloy et al. 2017; Shim et al. 2013).

Despite significant progress made by various institutions in the domain of neurodegenerative proteomics research, notable gaps remain in the understanding of proteomic regulation at the intersection of oxidative stress and neuroinflammation (Uddin et al. 2020). Current evidence unequivocally identifies oxidative stress as a pathogenic contributor to neurodegeneration; however, the intricate bidirectional interactions between oxidative modifications and neuroinflammatory signaling pathways are not yet fully delineated. Specifically, while it is established that oxidative stress mediates protein modifications and functional impairments through multiple pathways, the exact mechanisms by which these modifications initiate neuroinflammatory cascades remain ambiguous (Mu et al. 2024; LeFort et al. 2024), Additionally, although proteomic methodologies can effectively identify oxidatively modified proteins, the functional implications of these modifications within neuroinflammatory contexts have not been comprehensively explored. Consequently, there is an urgent need for future research to systematically investigate the interaction network between oxidative stress and neuroinflammation, as well as to elucidate the fundamental regulatory role of proteomics in this interplay.

The examination of authorship trends within the field has revealed a diverse network of contributors, comprising a total of 18,516 authors. Notably, Butterfield Da emerges as a prominent figure, having published 121 articles that collectively received over 13,488 citations, thereby underscoring his substantial influence on neurodegenerative proteomics research. This trend suggests that prolific authors have the capacity to shape the research agenda and foster collaboration across various subfields, thereby enhancing the collective comprehension of neurodegenerative mechanisms. Analyzing the dynamics of author collaborations and citation networks can provide significant insights into the communication and evolution of research within this scientific community (Alexander et al. 2017; Huang et al. 2015; Svider et al. 2013).

Journal analysis indicates that specialized publications are essential for sharing findings in the fields of proteomics and NDs. The *Journal of Proteome Research* leads with 149 articles, followed by the *Journal of Alzheimer's Disease* with 103 articles, and *Proteomics* with 87 articles. These journals not only provide platforms for significant research dissemination but also contribute to establishing scholarly standards and encouraging discussions on emerging topics in the field. The existence of high‐impact journals highlights the importance for researchers to choose suitable outlets for their work, ensuring maximum visibility and influence within the scientific community G. Huang et al. 2015; Vohra et al. 2022; Joksimovic et al. 2017). Notably, these journals have emerged as essential platforms for the dissemination of methodological innovations. Advanced computational methodologies are fundamentally altering biomarker discovery and disease stratification in neurodegenerative disorders (Reel et al. 2021). Deep neural networks are now capable of identifying intricate protein signatures that are often missed by traditional analytical methods, to uncover subtype‐specific biomarkers (Bryant 2023). Additionally, unsupervised learning techniques promote the identification of novel disease types by systematically analyzing complex protein expression signatures within multi‐dimensional omics data (Ge et al. 2024; Heo et al. 2025). This integration of proteomic technologies with machine learning is establishing new paradigms for the understanding and classification of these complex disorders (Sicilia et al. 2022; Agache et al. 2023).

The limitations of this study primarily stem from the inherent biases that come with bibliometric analyses. The reliability of the findings depends heavily on the quality and comprehensiveness of the data sources used; any gaps or inaccuracies in these databases can significantly impact the analysis's outcomes. Furthermore, bibliometric methods mainly concentrate on quantifiable metrics, such as the number of publications and citation frequencies, often overlooking the intrinsic quality of the literature. It is important to recognize that a highly cited article does not necessarily indicate high scientific merit. Additionally, the reasons for citations are complex and varied, ranging from agreement with the content to critical evaluation, which bibliometric methods are not designed to differentiate. Lastly, differences in publication volumes and citation practices across various disciplines may restrict the applicability of standard bibliometric indicators, potentially distorting the representation of different research areas.

In conclusion, this study highlights the significant role of proteomics in ND research, emphasizing its potential to enhance early diagnosis and develop new therapies despite certain limitations. Future research should integrate multi‐omics approaches for a comprehensive understanding of these diseases. While genomics has identified genetic variants linked to neurodegenerative conditions, proteomics can assess protein expression, modifications, and interactions, offering insights into disease mechanisms. (Li et al. 2021; Cruchaga et al. 2014; Wes et al. 2016). Additionally, transcriptomics, particularly single‐cell analyses, reveals gene expression patterns across various cell types and states (Tanke 2007; Suhre et al. 2017). The integrated analysis of proteomics and transcriptomics elucidates the mechanisms by which transcriptional modifications translate into alterations at the protein level, thereby enhancing our comprehension of the intricate regulation of gene expression (Fagerberg et al. 2014; Yadav et al. 2023). Furthermore, metabolomics investigates the dynamics of intracellular and extracellular metabolites, providing essential insights into metabolic dysregulation associated with NDs (Jové et al. 2014). The integration of proteomics with metabolomics enables researchers to elucidate the molecular mechanisms that underlie metabolic changes by examining the expression levels and activity states of metabolic enzymes, thus facilitating the identification of novel therapeutic targets (Iturria‐Medina et al. 2022; Gątarek and Kałużna‐Czaplińska 2024). Ultimately, the integration of proteomics with genomics, transcriptomics, and metabolomics will create a comprehensive framework for studying neurodegenerative disorders. This multi‐omics approach enables systematic analysis of genetic variants, protein functions, metabolic changes, and cellular phenotypes through unified data platforms that link molecular interactions to their pathological effects.

## Author Contributions

X.A. and J.H. wrote the manuscript. Z.R. and W.Y. designed the study. X.A. performed the data analysis and interpretation. J.H. contributed to the data collection. All authors have read and approved the final manuscript.

## Conflicts of Interest

The authors declare that no conflicts of interest.

## Data Availability

The authors have nothing to report.
